# Life-History Parameters of the Colorado Potato Beetle, *Leptinotarsa decemlineata*, on Seven Commercial Cultivars of Potato, *Solanum tuberosum*

**DOI:** 10.1673/031.013.13201

**Published:** 2013-11-23

**Authors:** Seyed Ali Asghar Fathi, Zoha Fakhr-Taha, Jabraeil Razmjou

**Affiliations:** Department of Plant Protection, University of Mohaghegh Ardabili, Ardabil, Iran

**Keywords:** Chrysomelidae, development, host plants, reproductive biology, suitability

## Abstract

The Colorado potato beetle, *Leptinotarsa decemlineata* (Say) (Coleoptera: Chrysomelidae), is an important pest of potato, *Solanum tuberosum* L. (Solanales: Solanaceae), crops in the Ardabil region of Iran. In this research, the life-history parameters of *L. decemlineata* were investigated on seven potato cultivars, namely Agria, Aozonia, Diamant, Cosmus, Kondor, Morene, and Savalan, in a greenhouse at 23 ± 1° C and 55 ± 5% RH under a natural photoperiod. The results indicated that the development time of larvae was longest on Savalan (18.3 days) among the tested potato cultivars. The survival rates (egg to adult) on Savalan and Morene were significantly lower than on the other studied cultivars. *L. decemlineata* reared on Savalan had the lowest fecundity (286.3 eggs/female) among the tested potato cultivars. The oviposition period of females was significantly shorter on Savalan and Diamant than on Kondor, Aozonia, Morene, Agria, and Cosmus. The values of intrinsic rate of natural increase and population growth rate were lowest on Savalan (0.055 and 1.056, respectively). The generation time and doubling time were significantly longest on Savalan (69.5 and 12.7 days, respectively). Based on these results, it can be concluded that Savalan is the least suitable cultivar for *L. decemlineata* among the ones tested. These results can be useful in integrated management of *L. decemlineata* in potato fields.

## Introduction

In the Ardabil region of northwest Iran, more than 25,000 ha of irrigated land is allocated for potato production (Anonymous 2009). The Colorado potato beetle, *Leptinotarsa decemlineata* (Say) (Coleoptera: Chrysomelidae), is the major insect pest of potato, *Solanum tuberosum* L. (Solanales: Solanaceae), crops in this region (Nouri-Ganbalani 1989; Nouri-Ganbalani et al. 2010). Both larvae and adults of *L. decemlineata* feed on the foliage of the potatoes, and uncontrolled populations can completely defoliate potato plants and reduce yield (Hare 1980; Weber 2003; Alyokhin 2008; Nouri-Ganbalani et al. 2010). In the Ardabil region, growers largely depend on insecticides for control of *L. decemlineata*, but it has developed resistance to many traditional organophosphate, carbamate, and pyrethroid insecticides (Mota-Sanchez et al. 2002). The inadequacy and the potential adverse effects on the environment of chemical use have prompted research efforts to find alternative approaches, such as host plant resistance, to control the pest (Smith 1989; Panda and Khush 1995; Weber 2003; Weber et al. 2006; Alyokhin 2008; Fréchette et al. 2010; Werling et al. 2012). *L. decemlineata* responds differently to various potato species and cultivars, and some reports have been published concerning the resistance of potato cultivars to *L. decemlineata* (Kennedy et al. 1987; Yaşar and Güngör 2005). Potato plants that exhibit antibiotic resistance to *L. decemlineata* can be effective in preventing the beetle population from reaching economic damage levels (Kennedy et al. 1987; Yaşar and Güngör 2005). Furthermore, potato plants with antibiosis mechanism may have an indirect management effect by increasing the exposure of *L. decemlineata* to its natural enemies as a result of prolonged developmental time. One method of selecting resistant cultivars is the study of the lifehistory parameters of *L. decemlineata*, especially the intrinsic rate of natural increase (*rm*). The value of *r_m_* is used by most ecologists as a comparative statistic to determine the effect of different treatments (host quality, temperature, etc.) on the development rate and reproductive capacity of an insect (Smith 1989; Carey 1993).

Some potato cultivars, namely Agria, Aozonia, Diamant, Cosmus, Kondor, Morene, and Savalan, have recently been introduced in the Ardabil region. Agria and Diamant have their origin in Germany, while Aozonia, Cosmus, Kondor, and Morene have their origin in the Netherlands, and Savalan is native to in Iran. The ancestor of these cultivars was *S. tuberosum*. All seven potato cultivars that were studied in this research were commercial varieties in the Ardabil region. Currently, Agria is being planted on a greater acreage than the other potato cultivars evaluated by this research. Field observations (2008 and 2009) indicated that these seven cultivars had the lowest populations of *L. decemlineata* among the potato cultivars in the Ardabil region. The literature indicated that some research has compared the life-history parameters of *L. decemlineata* on potato cultivars (Yaşar and Güngör 2005; Lyytinen et al. 2007), but the lifehistory parameters of *L. decemlineata* have not been studied on the above-mentioned potato cultivars**. Therefore, the purpose of this study was to compare the life-history parameters of *L. decemlineata* on seven potato cultivars currently in use to determine the most resistant cultivar, which could then be used in integrated management of this pest.

## Materials and Methods

### Insect colony

The laboratory colony of *L. decemlineata* was initiated from a collection made in a potato field (cv. Marphona) at the Agricultural Research Station of the University of Mohaghegh Ardabili in 2009. Stock cultures of *L. decemlineata* were established by rearing the collected eggs on potato foliage (cv. Marphona) for one generation in rearing units (20 cm in diameter and 20 cm in depth, fitted with mesh lids for ventilation) in a greenhouse at 23 ± 1° C, 50 ± 5% RH, and a natural photoperiod. Sawdust was placed on the bottom of each rearing unit as a site for pupae to develop. The potato foliage used for beetle rearing was previously disinfected in 2% sodium hypochlorite for 5 minutes. A pair of newly emerged adults was introduced into a oviposition container (12 cm in diameter and 15 cm in depth, fitted with mesh lids) that had five fresh leaves. After a 1-day oviposition period, the adult beetles were removed and the leaves with 1-day-old eggs were used in the life table experiments.

### Source plants

Tubers of tested cultivars, namely Agria, Aozonia, Diamant, Cosmus, Kondor, Morene, and Savalan, were obtained from the Seed and Plant Improvement Institute of Iran in Karaj, Iran. The tubers of tested cultivars were grown individually in 20-cmdiameter pots in a greenhouse at 23 ± 1° C and 55 ± 5% RH under a natural photoperiod. No insecticides were applied to the plants. All pots were soaked with the fungicide Dithane (Mancozeb; Rohm and Haas Co., Dow Chemical Company, www.dow.com) to prevent foliar diseases at the beginning of the stem elongation stage. In all experiments, plants were used after growing to the inflorescence emergence stage because potato plants at the inflorescence emergence stage are more susceptible to *L. decemlineata* damage (Shields and Wyman 1984; Zehnder and Evanylo 1989; Hare 1990; Zehnder et al. 1995).

### Life-table study

The life-history parameters of *L. decemlineata* were studied on seven potato cultivars in a greenhouse at 23 ± 1° C and 50 ± 5% RH under a natural photoperiod using plastic clip cages (12 cm diameter and 15 cm depth, fitted with mesh lids) that were established on nearly 7-day-old leaves of each potted plant (N = 10 for each cultivar). One-day-old eggs taken from the cultivars were individually glued (using 50% honey solution) to the lower surface of a predetermined leaf of the potted plant from the same cultivar, and each leaf containing an egg was confined with a clip cage. Two or three clip cages were established on each plant, and each clip cage was considered a replicate (N = 24 for each of seven studied cultivars). These experiments were conducted in a completely randomized design. Daily observations were made to determine the egg-hatching time, larval development time, and survival rate during immature stages. After completing development of the last instar larvae inside the cage, the sawdust was placed on the bottom of each clip cage (3 cm depth) as a site for pupae to develop until adult emergence. Adults were then sexed, and the number of females and males was recorded on each cultivar. The sex of emerged adults on each cultivar was determined by examining the ventral tip of the abdomen; the posterior end of the last ventral abdominal segment is depressed in the male, whereas in the female this depression is absent (Rivnay 1928).

To determine fecundity on each cultivar, a pair of beetles was transferred to an oviposition clip cage (12 cm diameter and 15 cm depth, fitted with mesh lids) that was established on a nearly 7-day-old leaf of the potted plant from the corresponding cultivar. The leaf along with the cage was removed daily, and the number of eggs deposited on the leaf and cage was counted using a hand lens (10 X). A pair of beetles was then transferred to a new clip cage established on another leaf of the same potted plant. If a female beetle died within the first 24 hr, it was replaced with a newly emerged beetle. This study was continued until the death of female and male beetles in all cages. In this experiment, the age-specific fecundity of *L. decemlineata* was recorded on each potato cultivar.

### Data analysis

Prior to analysis, data of the life-history parameters were log-transformed to correct for heterogeneity of variance, whereas data on survival rate were arcsine-transformed. Data for the life-history parameters were analyzed using one-way ANOVA. The differences among treatment means were compared using Student-Newman-Keuls test or Tukey's HSD test (SAS Institute 2005). The following equation was applied to calculate the intrinsic rate of natural increase (*r_m_*) of *L. decemlineata* (Birch 1948; Laughlin 1965):

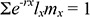
Where *e* is the base of natural logarithms, *x* is the age of the immature and mature stages in days, *l_x_* is survival of the immature and mature stages until *x*, and *m_x_* is the number of female offspring for a specific age *x*.

**Table 1.**
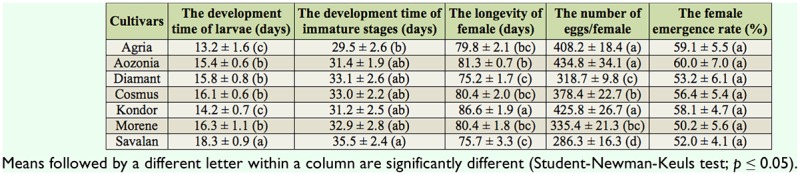
Mean (SE) egg-hatching time, larval development time, pupal development time, longevity of females, fecundity, and sex ratio of *Leptinotarsa decemlineata* reared on seven potato cultivars.

The jackknife technique was used to compare the *r_m_* values and the other life table parameters on seven potato cultivars (Maia et al. 2000). After calculating *r_i_′* for *n_i_′* series data and *r_m_* for the original data (*r_all_*), the jackknife pseudo-value (*r_j_*) was computed for the *n* samples by using the following formula:

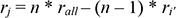
The jackknife pseudo-value (*r_j_*) for each cultivar was analyzed using one-way ANOVA, and the differences were compared using Tukey's HSD test (SAS Institute 2005).

## Results

There were significant differences among the cultivars with respect to the development time of larval stages (df = 6, 97; F = 18.25; *p* = 0.0001) and immature stages (df = 6, 44; F = 11.66; *p* = 0.0001) of *L. decemlineata*. The egg-hatching time (df = 6, 133; F = 0.97; *p* = 0.45) and duration of pupal stage (df = 6, 44; F = 0.66; *p* = 0.682) were not statistically significant on the seven potato cultivars (Table 1). The development times of larvae and immature stages on Savalan were longer compared to the other studied cultivars. The development times of larval stages on Morene, Cosmus, Diamant, and Aozonia were significantly longer than on Agria and Kondor (Table 1).

**Table 2.**
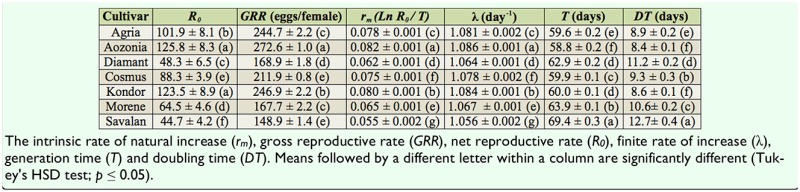
Life table parameters of *Leptinotarsa decemlineata* reared on seven potato cultivars.

**Figure 1.**
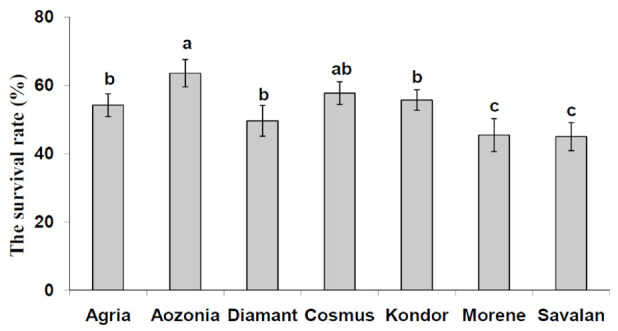
Mean (± SE) survival rate of *Leptinotarsa decemlineata* reared on seven potato cultivars (Student-Newman- Keuls test; *p* ≤⃒ 0.05). y-axis = % survival. High quality figures are available online.

The longevity and fecundity of *L. decemlineata* were statistically significant when they were reared as larvae on the tested cultivars of potato (df = 6, 26; F = 6.98; *p* = 0.0002; df = 6, 26; F = 12.31; *p* = 0.0001) (Table 1). The longevities of females on Savalan (75.7 days) and Diamant (75.2 days) were significantly shorter compared to Kondor and Aozonia, but were significantly different among Savalan, Diamant, Agria, Cosmus, and Morene (Table 1). The lowest fecundity of females was observed when they were reared as larvae on Savalan (286.3 eggs/female). The fecundity of beetles decreased on the cultivars in the following order: Aozonia, Kondor, Agria, Cosmus, Morene, Diamant, and Savalan (Table 1).

Potato cultivars had a significant impact on the survival rate (egg to adult) of *L. decemlineata* (df = 6, 26; F = 6.82; *p* = 0.0002) (Figure 1). The survival rates on Savalan (45%) and Morene (45.5%) were significantly lower than on Aozonia and Cosmus. For all the potato cultivars, the survival rates decreased in the following order: Aozonia, Cosmus, Agria, Kondor, Diamant, Morene, and Savalan (Figure 1). The female emergence rates of *L. decemlineata* reared as larvae on the seven tested cultivars were between 50.2 and 60.0%. The female emergence rates were not statistically significant among the tested cultivars (df = 6, 26; F = 7.01; *p* = 0.0002).

The population growth parameters of *L. decemlineata* reared on the tested potato cultivars are given in Table 2. The intrinsic rate of natural increase (*r_m_*) (df =6, 96; F = 455.71; *p* = 0.0001), net reproductive rate (df = 6, 96; F = 208.63; *p* = 0.0001), generation time (df = 6, 96; F = 2981.59; *p* = 0.0001), doubling time (df = 6, 96; F = 354.56; *p* = 0.0001), and population growth rate per day (df = 6, 96; F = 456.68; *p* = 0.0001) of *L. decemlineata* varied significantly among the studied cultivars (Table 2). The value of *r_m_* on Savalan (0.055) was significantly lowest among the tested cultivars. The respective descending order of *r_m_* values was Aozonia, Kondor, Agria, Cosmus, Morene, Diamant, and Savalan. The net reproductive rate on Aozonia, Kondor, Agria, Cosmus, Morene, and Diamant were about 2.81, 2.76, 2.28, 1.97, 1.44, and 1.08 times the value of that reared on Savalan, respectively. The generation time was longest on Savalan (69.4 days) and shortest on Aozonia (58.8 days). The doubling time of the population reared on Savalan (12.7 days) was significantly longer compared to the other cultivars tested. The finite rate of population growth was lowest on Savalan (1.056) and highest on Aozonia (1.086), and for all potato cultivars the finite rate of population growth decreased in the following order: Aozonia, Kondor, Agria, Cosmus, Morene, Diamant, and Savalan (Table 2).

## Discussion

Our study demonstrated that the type of potato cultivars had a significant effect on the development, survival, and reproduction of *L. decemlineata*. Beetles reared on Savalan cultivar had the lowest fecundity (286.3 eggs/female) and the longest larval development time (18.3 days). The development time of immature stages of *L. decemlineata* was 29.5 and 35.5 days on Agria and Savalan, respectively. The survival rate (egg to adult) was estimated to be 45 and 63.6% on Savalan and Aozonia, respectively. The slower development rate and lower survival rate and fecundity of *L. decemlineata* on Savalan would result in lower population growth, which in turn should lead to lower subsequent infestations by this pest. The findings of our study are consistent with earlier reports. For example, Yaşar and Güngör (2005) demonstrated that the development time of immature stages of this beetle ranged from 31.3 to 35.9 days on five potato cultivars. Furthermore, they demonstrated that the survival rate of *L. decemlineata* (immature stages except eggs) was significantly different among the five tested potato cultivars and ranged between 21.81 and 71.43% depend on the potato cultivars. However, Lyytinen et al. (2007) concluded that the survival rate of larvae of *L. decemlinata* was not significantly different among three potato cultivars. In our research, the fecundity of *L. decemlinata* was 408.2 eggs/female on Agria, which is 1.34 times greater than that reported by Yaşar and Güngör (2005). Yaşar and Güngör (2005) reported that the fecundity of this beetle ranged between 303.7 and 507.0 eggs/female on Agria and Pasinler, respectively.

Because the intrinsic rate of natural increase is a reflection of many factors, such as fecundity, survival, and generation time, it adequately summarizes the physiological qualities of an animal in relation to the animal's capacity to increase population. Therefore, it is the most appropriate index to evaluate the performance of an insect on different host plants as well as the host plant's resistance (Smith 1989; Carey 1993; Southwood and Henderson 2000). In our research, the intrinsic rates of natural increase of *L. decemlineata* were statistically significant among the tested potato cultivars. The lowest and highest *r_m_* values were obtained on Savalan and Aozonia (0.055 and 0.082, respectively). The *r_m_* value of *L. decemlineata* on the plant species and/or cultivars within the same species is influenced by the morphological and biochemical characteristics of the host plants (Lyytinen et al. 2007; Fréchette et al. 2010). The findings of our study are consistent with earlier reports. For example, Yaşar and Güngör (2005) concluded that the *r_m_* values of *L. decemlineata* were 0.028 and 0.051 on Granola and Pasinler, respectively. The lowest *r_m_* value being on Savalan indicates that this cultivar was the least suitable cultivar among the tested cultivars of potato. Savalan cultivar had the highest antibiosis resistance against *L. decemlineata*, as indicated by the slower development rate, lower survival rate, and lower reproduction compared to the other cultivars. Consequently, it may be concluded that the *L. decemlineata* population could increase at a lower rate on the Savalan (finite rate of increase = 1.056) cultivar compared to the other tested cultivars of potato. Based on direct observation of tested cultivars leaves under a stereomicroscope, it was clear that leaf trichomes (grandular and simple) on Morene, Diamant, and Agria cultivars were higher compared to the other cultivars. The lowest leaf trichomes were observed on Savalan. One prominent characteristic of Savalan cultivar is more vegetative growth. Several factors, such as leaf trichomes and leaf biochemistry of potato host plants, affect survival rate and population growth of *L. decemlineata*, as has been reported by previous research (Mattson 1980; Hare 1987; Clancy 1992; Awmack and Leather 2002; Lyytinen et al. 2007; Fréchette et al. 2010). Further research is needed to investigate the morphological and biochemical characteristics of the tested potato cultivars.

The use of resistant cultivars has an important role in the integrated management of *L. decemlineata* in Iran. However, the resistance level of the Savalan cultivar (survival rate = 45%, *r_m_* value = 0.055, and doubling time = 12.7 days) reported in this paper is not sufficient to achieve adequate control of *L. decemlineata* by itself. Therefore, further research is required to investigate the potential of using the Savalan cultivar in combination with other control tactics in the integrated management of this pest.

## Abbreviations:

*rm*,: intrinsic rate of natural increase
